# Medial Laminectomy of the Large Concha Bullosa in Crooked Nose

**Published:** 2018-01

**Authors:** Fatih Ozdogan, Halil Erdem Ozel, Erkan Esen, Selahattin Genc, Adin Selcuk

**Affiliations:** Derince Education and Research Hospital ENT Clinic, Kocaeli/ Turkey

**Keywords:** Rhinoplasty, Laminectomy, Concha Bullosa, Crooked Nose

**DEAR EDITOR**

Crooked or deviated nose is one that varies from the straight vertical orientation of the face. Crooked nose is associated with nasal obstruction, sinus headaches and sinus infections. It is essential the external nasal frame, and the internal nasal structures such as nasal valve, and turbinates in crooked nose.^1^^,^^2^ Middle turbinate pneumatization or concha bullosa (CB) is one of the most common anatomical variations of the middle meatus.^3^ It has been reported that there is a relationship between septum deviation (SD) and CB in the literature.^4^

Therefore, there is a relationship between crooked nose and CB, too and because of that, intervention for the concha is important in crooked nose cases. There are many methods have been described for the surgical treatment of the CB: crushing, turbinoplasty and partial resection. Partial resection methods are medial and lateral laminectomy.^5^ The aim of the medial laminectomy of the large CB technique in the crooked nose is the prevention of contact of medial lamina of the middle turbinate and ethmoid perpendicula plate, and the nasal axis deviation in the postoperative period.

**Fig. 1:**
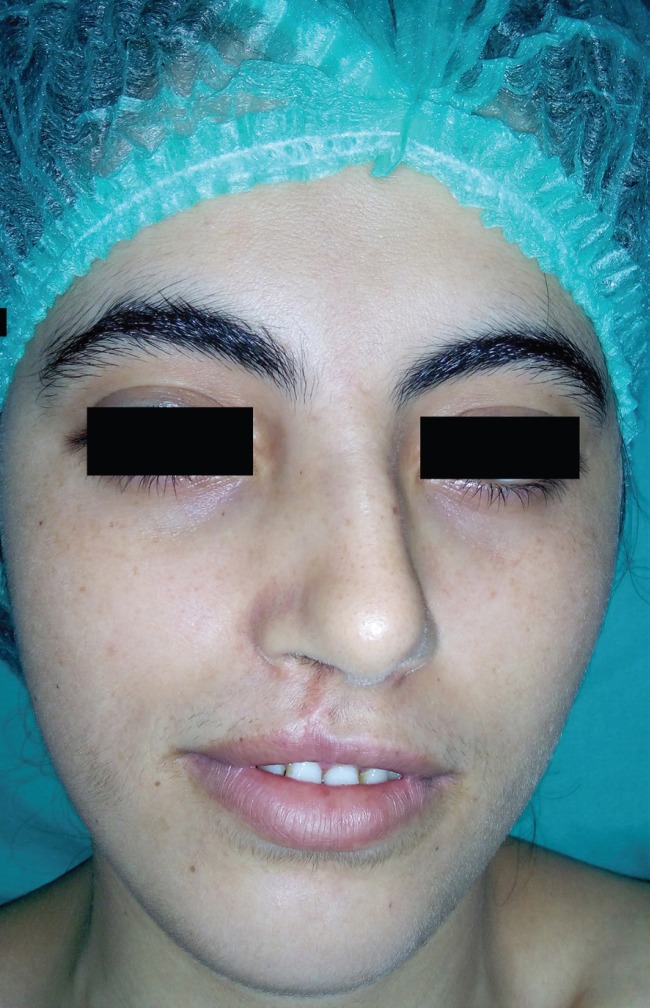
Preoperative photo.

**Fig. 2:**
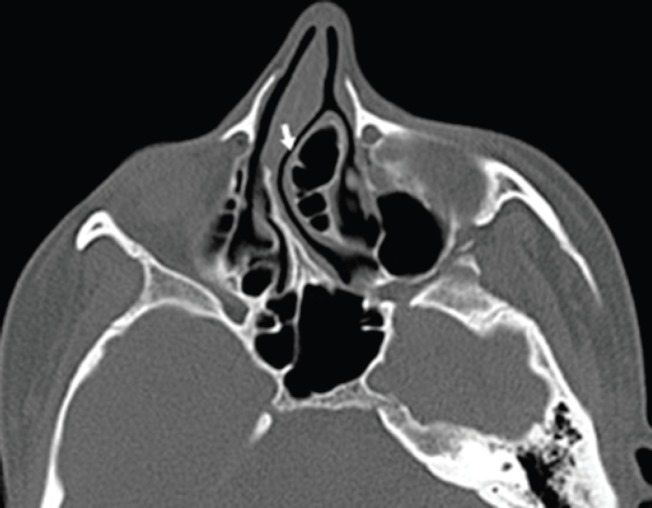
Preoperative axial paranasal sinus computerized tomography scan (white arrow: left medial wall of pneumatized middle turbinate).

This technique (medial laminectomy of the middle turbinate) has been performed on five patients (3 females, 2 males). All patients had crooked nose and one side large extended CB (Figure 1 and 2). The average age of patients was 24 years (19-35 years). The average follow-up duration was 6 months (4-7 months). Postoperative major complications were not noted in any on the patients. In one patient, minimally medial mucosal adhesion of the middle turbinate was seen and following, surgical intervention was not performed. Hyposmia and nasal obstruction were not seen in any patients. A Nasal Obstruction Symptom Evaluation (NOSE) questionnaire was used to evaluate disease-specific quality of life in all patients.^6^ The NOSE scores were significantly decreased after the operation in all patients. 

**Fig. 3:**
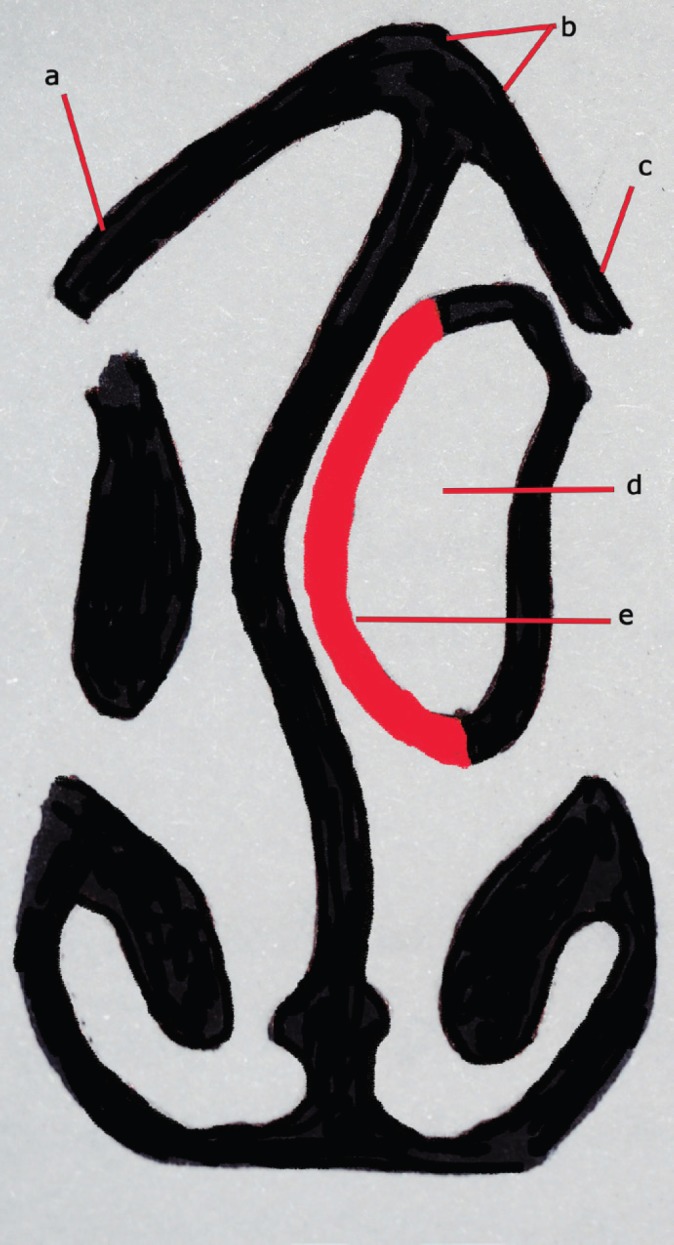
An illustration showing the preoperative left middle turbinate pneumatization in crooked nose and operation plan (a: right double lateral osteotomy, b: paramedian osteotomies, c: left lateral osteotomy, d: pneumatized middle turbinate, e: medial wall of pneumatized middle turbinate).

**Fig. 4:**
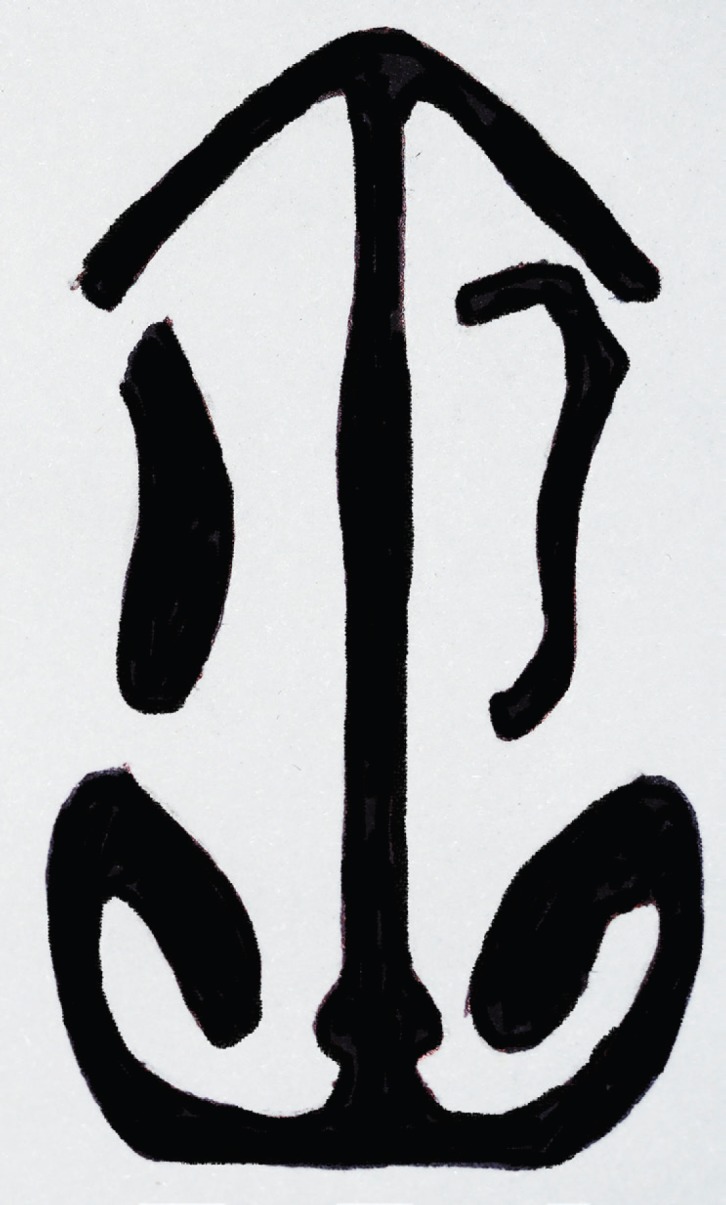
An illustration showing the postoperative image.

**Fig. 5:**
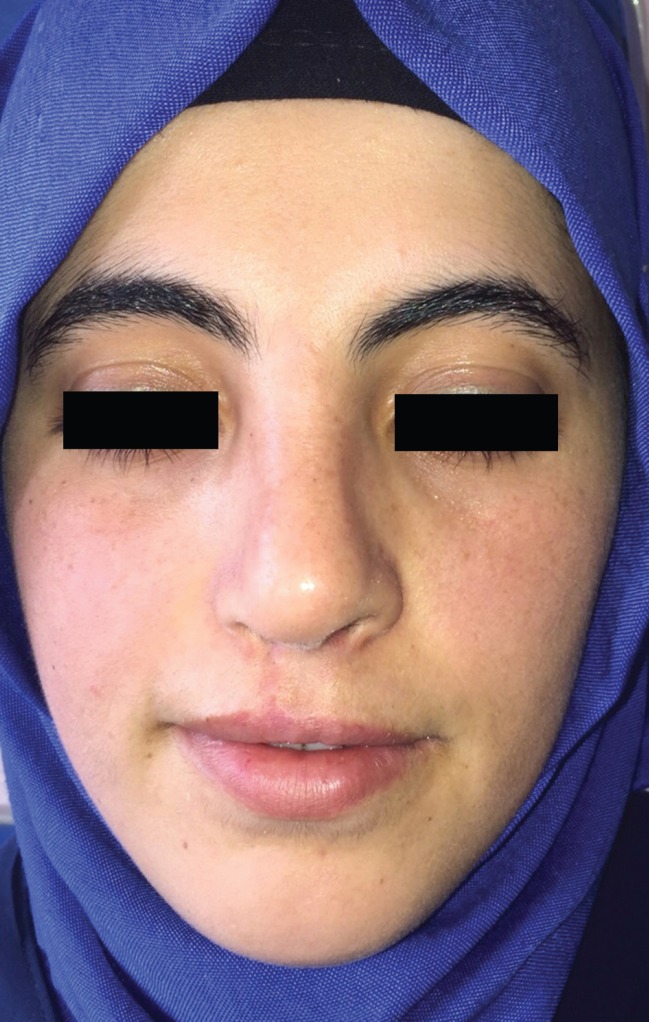
Postoperative photo.

**Fig. 6:**
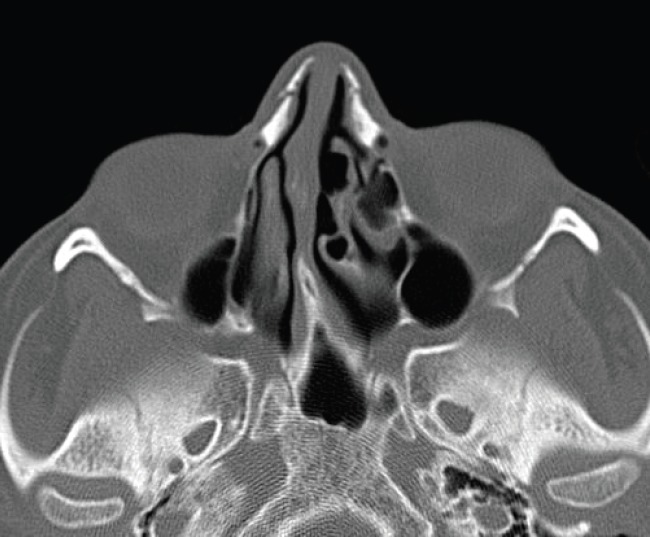
Postoperative axial paranasal sinus computerized tomography scan.

The surgical procedure was performed under general anesthesia. Middle turbinate surgery was performed before septorhinoplasty. The 0-degree rigid telescope was used for the procedure. Intranasal lidocaine (20 mg/ml) and epinephrine HCl (0.0125 mg) were used for local anesthesia and vasoconstriction. A midline vertical incision of the middle turbinate with a sickle knife, medial lamina of the turbinate was cut out with a turbinate scissor and Blakesley nasal forceps (Figure 3 and 4). Then, open septorhinoplasty was performed and nasal axis deviation was corrected (Right double osteotomy, paramedian osteotomy and left single lateral osteotomy were performed in this case, Figure 5 and 6).

Middle turbinate pneumatization is one of the most common anatomical variations of the middle meatus.^3^ Three types of middle turbinate pneumatization were evident. In the first type, air cells were noted to pneumatize the vertical lamella of the turbinate. In the second type, air cells were noted to pneumatize the inferior or bulbous segment of the turbinate. In the third type, extensive pneumatization of the lamellar and bulbous portion of the turbinate.^7^ The incidence of middle turbinate pneumatization in the literature ranges from 14% to 53%.^8^

Extensive pneumatization of the middle turbinate has been implicated as a possible etiologic factor in nasal obstruction, recurrent sinusitis and headache.^5^^,^^7^ Several techniques have been described for the surgical treatment of middle turbinate pneumatization; crushing, turbinoplasty and partial resection.^5^ Crushing the middle turbinate does not affect the physiology or anatomy or lead to recurrence. However, crushing is not applied for large middle turbinate pneumatization, which requires partial resection. Partial resection methods are medial and lateral laminectomy. Medial laminectomy is not popular because of medial mucosal adhesions. Medial adhesions of the middle turbinate are not a problem for surgeons and are sometimes even desirable. There is no detectable adverse effect on the sense of smell after medial laminectomy of the middle turbinate.^9^

Radiographic imaging is usually not a standard part of the work-up in patients interested in rhinoplasty. However, it can be helpful in patients who may benefit from concurrent turbinate surgery. Imaging can identify anatomic variations of the nasal bones and turbinates.^10^ We think for the patients with crooked nose, even if they do not have concomitant diseases (allergy, chronic sinusitis, etc.), preoperative CT is required. The crooked or twisted nose results from a complex deformity of the bony pyramid, the upper and lower cartilaginous vaults, and the septum and causes functional and aesthetic problems.^11^ The crooked nose’s major component is the extremely deviated nasal septum. Therefore, in order to correct the crooked nose, the nasal septum must be the treatment’s target.^12^

Medial lamina of the large pneumatized middle turbinate may cause the deviation of nasal axis by prompting the dorsal septal cartilage and perpendicular plate of etmoidal bone especially on dorsal and caudal portions following rhinoplasty operation. Even if medial laminectomy for the large CB has been a defined method previously, ıt is rarely applied in the literature. So, we think that if there is a togetherness of crooked nose and large CB, medial laminectomy will be a more promising choice.
